# Laboratory risk factors for coexistent primary biliary cholangitis in patients with Sjögren’s syndrome: a retrospective study

**DOI:** 10.1186/s12876-023-02859-4

**Published:** 2023-06-26

**Authors:** Xuan Gao, Guangzhi Xiao, Fengfan Yang, Rongrong Dou, Miao Xue, Yingying Zhang, Zhaohui Zheng, Jin Ding

**Affiliations:** Department of Clinical Immunology, Xijing Hospital, Fourth Military Medical University, No. 127 Changle West Rd., Xi’an, 710032 Shaanxi, China

**Keywords:** Retrospective study, Primary biliary cholangitis, Sjögren’s syndrome, Risk factors, Coexistence

## Abstract

**Background:**

Limited research exists on the laboratory characteristics of coexistent primary biliary cholangitis (PBC) and Sjögren’s syndrome (SS). This study aimed to investigate the laboratory risk factors for the coexistence of PBC in patients with SS.

**Methods:**

Eighty-two patients with coexistent SS and PBC (median age 52.50 years) and 82 age- and sex-matched SS controls were retrospectively enrolled between July 2015 and July 2021. The clinical and laboratory characteristics of the two groups were compared. Laboratory risk factors for the coexistence of PBC in patients with SS were analyzed using logistic regression analysis.

**Results:**

Both groups had a similar prevalence of hypertension, diabetes, thyroid disease, and interstitial lung disease. Compared with the SS group, patients in the SS + PBC group had higher levels of liver enzymes, immunoglobulins M (IgM), G2, and G3 (P < 0.05). The percentage of patients with an antinuclear antibody (ANA) titre > 1:10000 in the SS + PBC group was 56.1%, higher than that in the SS group (19.5%, P < 0.05). Additionally, cytoplasmic, centromeric, and nuclear membranous patterns of ANA and positive anti-centromere antibody (ACA) were observed more frequently in the SS + PBC group (P < 0.05). Logistic regression analysis showed that elevated IgM levels, high ANA titre, cytoplasmic pattern, and ACA were independent risk factors for PBC coexistence in SS.

**Conclusions:**

In addition to established risk factors, elevated IgM levels, positive ACA, and high ANA titre with cytoplasmic pattern provide clues to clinicians for the early screening and diagnosis of PBC in patients with SS.

## Background

Sjögren’s syndrome (SS) is a chronic autoimmune disease characterized by dry mouth and eyes and is caused by lymphocyte infiltration of the exocrine glands [1]. Liver involvement is considered the most common non-exocrine feature in SS with an incidence of 9–20% [2], and in approximately 3–9% of patients, primary biliary cholangitis (PBC) coexist with SS [3, 4]. PBC, a progressive cholestatic liver disease characterized by the destruction of small intrahepatic bile ducts, can lead to fibrosis and potential cirrhosis through its complications [5]. The combination of PBC and SS is undeniably associated with a poor prognosis. Therefore, early detection and diagnosis of PBC are of great significance for patients with SS.

PBC is diagnosed based on clinical, laboratory, and morphological criteria. Although a high alkaline phosphatase (ALP) level, in conjunction with the presence of an anti-mitochondrial antibody (AMA), is sufficient to diagnose this disorder, some problems still exist for the early diagnosis of PBC in patients with SS. The levels of serum cholestasis markers (i.e., ALP and γ-glutamyl transferase [GGT]) have been reported to be lower in patients with PBC with SS than in patients with no signs of SS [6], which can be overlooked in clinical practice. The prevalence of AMA-negative PBC in SS (22%, [3]) was higher than the commonly reported prevalence of AMA-negative PBC of approximately 5–10% [7]. The classical symptoms of PBC, which include fatigue and pruritus in the early phase, are non-specific. Reportedly, the diagnosis of PBC is established in the absence of symptoms in many cases (20–60%), and the proportion of asymptomatic cases at diagnosis has been increasing in recent years [8]. Thus, in patients with SS, paticularly those without symptoms, it is important to precisely identify the risk factors for PBC, such as autoimmune liver disease-related autoantibodies (i.e., anti-Sp100 and anti-gp210), which would be helpful for early PBC diagnosis.

However, most previous studies have focused on the differences between comorbid PBC and SS and PBC alone [9]. Limited research exists on the laboratory characteristics of comorbid PBC and SS compared with those of SS. Therefore, this retrospective study aimed to analyse the clinical and laboratory characteristics of the two patient groups to investigate other related risk factors for PBC in the SS group, in addition to established factors such as ALP and AMA.

## Methods

This was a retrospective observational study.

### Patients

In total, we included 103 patients who attended our outpatient and inpatient services from July 2015 to July 2021 and fulfilled the following SS and PBC criteria. The diagnosis of SS was based on the 2002 American-European Consensus Group criteria [10]. The diagnosis of PBC was based on the 2017 European Association for the Study of Liver Diseases criteria [11]. A diagnosis of SS combined with PBC was established if the patients simultaneously fulfilled the two sets of criteria forementioned. The exclusion criteria were patients diagnosed with other autoimmune diseases, including rheumatoid arthritis (RA), systemic lupus erythematosus (SLE), myositis, and malignant tumors. Patients with drug-induced alterations, alcoholic liver disease, or viral infection were also excluded. In the SS + PBC group, an overlap PBC/autoimmune hepatitis syndrome was also excluded as previously described [12]. Sex- and age-matched patients with SS during the same period were randomly selected as the control group at a ratio of 1:1.

### Data collection

Clinical observations were recorded at the patient’s initial visit, including age, sex, complications, and occurrence of cirrhosis. Laboratory findings were obtained, including liver function tests (Hitachi 7600 automatic biochemical analyser, Japan), complete blood counts (Sysmex XN-1000, Japan), and erythrocyte sedimentation rate (ESR). Immunological indices were determined, including serum immunoglobulins (Ig), complement 3 (C3), complement 4 (C4) (immunoturbidimetry, Beckman Coulter, IMAGE 800), and IgG subclasses (immunoturbidimetry, Siemens BN Pro-Spec). The ANA testing was detected by indirect immunofluorescent (IIF) on Hep-2 and liver tissue (monkey) substrate (Euroimmun AG, Germany) in dilution of 1:100, 1:320, 1:1000, 1:3200, 1:10000, > 1:10000. The slides were evaluated by two experienced laboratory professionals independently. The ANA fluorescence patterns were categorized based on the international consensus on standardized nomenclature of ANA HEp-2 cell patterns. Anti-nRNP/Sm, Sm, SSA, Ro-52, SSB, Scl-70, PM-Scl, PCNA, Jo-1, CENP-B, nucleosomes, histones, ribosomal protein-P, and anti-mitochondrial antibodies (AMA-M2) were detected by line immunoassay (LIA) (Euroimmun AG, Germany). The samples were also analyzed for autoantibodies with IIF (including AMA, anti-LKM, ASMA) (EuroImmun AG, Germany) and the Euroline Profile Autoimmune Liver Diseases (IgG) LIA (EuroImmun AG, Germany), which detects following antibodies: AMA-M2, M2-3E, Sp100, PML, Gp210, LKM-1, LC-1, SLA/LP and Ro-52. This study was approved by the ethics committee of our hospital. The data used in this study were obtained from the Chinese Rheumatology Cohort Database of the Department of Clinical Immunology in our hospital, and informed consent was obtained from the patients when their data were added in the database.

### Statistical analysis

Demographic variables were shown as mean ± standard deviation or median (interquartile range [IQR]), wherever applicable. Categorical variables were expressed as numbers (%). Intergroup comparisons were performed using Student’s t-test or a nonparametric test (Mann–Whitney U test) for continuous variables and the χ^2^ test or Fisher’s exact test for categorical variables. Based on biological plausibility and a literature review, multivariable logistic regression was used to analyse the risk factors of the combination of SS and PBC. The predictive value of the different risk indicators was estimated using the receiver operating characteristic (ROC) curve, reporting the area under the curve (AUC) and its confidence interval (Cl). Statistical analyses were performed using the SPSS software package (version 22.0, IBM Corp., Armonk, NY, USA), and statistical significance was set at P < 0.05.

## Results

### Patient characteristics

The study enrolled 82 patients with SS complicated by PBC (SS + PBC) and 82 patients with SS as the control group. Twenty-one patients were excluded, including eight with SLE, one with RA, two with autoimmune hepatitis, and 10 with incomplete data. The baseline variables are presented in Table 1. In the SS + PBC group, 46 of 82 patients underwent labial salivary gland biopsies and 17 patients underwent liver biopsy. In the SS group, 55 of 82 patients underwent labial salivary gland biopsy. As shown in Table 1, no significant differences occurred in sex, age, or clinical comorbidities (hypertension, diabetes, thyroid disease, interstitial lung disease, and hematologic involvement; P > 0.05). Patients in the SS + PBC group had a higher frequency of liver cirrhosis than those in the SS group (P < 0.001). In the SS + PBC group, 21 patients developed liver cirrhosis within 0 to 9 years, with an average of 3.53 ± 2.56 years. Among them, three patients already had liver cirrhosis at their first visit. Conversely, the SS group had only three patients who developed liver cirrhosis, with two being diagnosed with liver cirrhosis at their initial visit and one progressing to liver cirrhosis after two years of diagnosis.

**Table 1:** Comparison of the demographic and laboratory data between the SS only and SS + PBC groups

Variables	SS group (n = 82)	SS + PBC group (n = 82)	P value
Female (n, %)	77 (93.9)	79 (96.3)	0.720
Age (years, mean ± SD)	50.61 ± 12.97	53.9 ± 10.79	0.079
Duration of disease (months, median (IQR))	48(24,96)	36(12, 84)	0.117
Hypertension (n, %)	14 (17.1)	11 (13.4)	0.525
Diabetes (n, %)	5 (6.1)	8 (9.8)	0.386
Thyroid disease (n, %)	17 (20.7)	17 (20.7)	1.000
ILD (n, %)	32 (39.0)	21 (25.6)	0.066
Liver cirrhosis (n, %)	3 (3.7)	21 (25.6)	0.000*
Hematologic involvement (n, %)	28 (34.1)	27 (32.9)	0.869
Elevated ALT (n, %)	9 (11.0)	39 (47.6)	0.000*
Elevated AST (n, %)	16 (19.5)	49 (59.8)	0.000*
Elevated TBIL (n, %)	5 (6.1)	15 (18.3)	0.017*
Elevated DBIL (n, %)	10 (12.2)	21 (25.6)	0.028*
Elevated ALP (n, %)	7 (8.5)	41 (50.0)	0.000*
Elevated GGT (n, %)	17 (20.7)	66 (80.5)	0.000*
Decreased WBC (n, %)	13 (15.9)	19 (23.2)	0.237
Decreased hemoglobin (n, %)	30 (36.6)	27 (32.9)	0.623
Decreased platelet (n, %)	21 (25.6)	33 (40.2)	0.046*

*: P < 0.05.

SD, standard deviation; IQR, interquartile range; ILD, interstitial lung disease; ALT, alanine aminotransferase; AST, aspartate aminotransferase; TBIL, total bilirubin; DBIL, direct bilirubin; ALP, alkaline phosphatase; GGT, γ-glutamyl transpeptidase; WBC, white blood cell; SS, Sjögren’s syndrome; PBC, primary biliary cholangitis.

### Comparison of features of liver function tests and complete blood counts between patients with SS only and those with SS + PBC

As shown in Table 1, the levels of biochemical indices (serum levels of alanine aminotransferase [ALT], aspartate aminotransferase [AST], total bilirubin [TBIL], direct bilirubin [DBIL], ALP, and GGT) were significantly higher in the SS + PBC group than in the SS group, while platelets levels were lower in the SS + PBC group than in the SS group (P < 0.05). No differences in white blood cell (WBC) count, or hemoglobin levels were observed between the two groups (P > 0.05).

### Comparison of immunoglobulin, complement, and IgG subclasses between patients with SS only and those with SS + PBC

The patients in the SS + PBC group had a higher frequency of increased immunoglobulin M (IgM) levels than those in the SS group [53.7% (44/82) vs. 6.1% (5/82), P < 0.001]. As shown in Table 2, compared with the SS group, the SS + PBC group had lower C4 and IgG1/IgG levels and higher levels of IgG2, IgG3, IgG2/IgG, and IgG3/IgG (P < 0.05).

**Table 2:** Comparison of immunoglobulin, complement, and IgG subclasses between patients with SS only and those with SS + PBC

Variables	SS group (n = 82)	SS + PBC group (n = 82)	P value
ESR (mm/h, median (IQR))	25.50 (11.00,49.25)	29.50 (17.00,45.50)	0.190
Elevated RF (n, %)	48 (58.5)	40 (48.8)	0.210
IgA (g/L, median (IQR))	2.97 (1.83,4.03)	2.86 (2.20,4.31)	0.836
IgG (g/L, median (IQR))	18.85 (14.28,25.53)	17.65 (13.95,23.00)	0.739
IgM (g/L, median (IQR))	1.18 (0.91–1.64)	2.94 (1.40–4.20)	0.000*
C3 (g/L, median (IQR))	0.76 (0.64,0.92)	0.84 (0.69,1.04)	0.032*
C4 (g/L, median (IQR))	0.18 (0.13,0.21)	0.15 (0.12,0.18)	0.009*
IgG1 (g/L, median (IQR)) †	10.35 (7.55,15.45)	10.45 (7.90,14.35)	0.926
IgG2 (g/L, median (IQR)) †	3.09 (2.44–3.96)	3.89 (2.86–5.79)	0.005*
IgG3 (g/L, median (IQR)) †	0.47 (0.30–0.74)	0.61 (0.41–1.13)	0.021*
IgG4 (g/L, median (IQR)) †	0.25 (0.14,0.49)	0.28 (0.17,0.44)	0.978
IgG1/ IgG (mean ± SD) †	0.71 ± 0.10	0.67 ± 0.11	0.024*
IgG2/ IgG (mean ± SD) †	0.22 ± 0.09	0.26 ± 0.09	0.047*
IgG3/ IgG (median (IQR)) †	0.03 (0.02,0.05)	0.04 (0.03,0.06)	0.009*
IgG4/ IgG (median (IQR)) †	0.02 (0.01,0.03)	0.02 (0.01,0.03)	0.524

*: P < 0.05.

†: Sixty-eight patients in the SS group and forty-eight patients in the SS + PBC group underwent immunoglobulin G subclass tests.

IgG, Immunoglobulin G; SS, Sjögren’s syndrome; PBC, primary biliary cholangitis; ESR, erythrocyte sedimentation rate; RF, rheumatoid factor; IQR, interquartile range; SD, standard deviation.

### Comparison of immunological indices between patients with SS only and those with SS + PBC

As shown in Table 3, ANA titres greater than 1:10000 were more common in the SS + PBC group (P < 0.05). In addition, cytoplasmic, centromeric, and nuclear membranous patterns were more frequently observed in the SS + PBC group, whereas the speckled pattern was more frequently observed in the SS group (P < 0.05). A significantly greater number of patients in the SS + PBC group were positive for ACA, AMA-M2, AMA, anti-3E, and anti-gp210 antibodies (P < 0.05) than those in the SS-only group, whereas no significant difference was observed for other antibodies.

**Table 3:** Comparison of serum autoantibodies between patients with SS only and those with SS + PBC

Variables (n, %)	SS group (n = 82)	SS + PBC group (n = 82)	P value
ANA title > 1:10000	16 (19.5)	46 (56.1)	0.000*
Speckled	67 (81.7)	43 (52.4)	0.000*
Homogeneous	6 (7.3)	4 (4.9)	0.514
Cytoplasmic	15 (18.3)	70 (85.4)	0.000*
Centromeric	4 (4.9)	23 (28)	0.000*
Nucleolar	3 (3.7)	2 (2.4)	1.000
Nuclear membranous	0 (0)	7 (8.5)	0.014*
Nuclear dot	1 (1.2)	1 (1.2)	1.000
Anti-SSA	52 (63.4)	45 (54.9)	0.266
Anti- Ro52	64 (78.0)	60 (73.2)	0.467
Anti-SSB	20 (24.4)	18 (22.0)	0.711
ACA	5 (6.1)	26 (31.7)	0.000*
AMA-M2	2 (2.4)	54 (65.9)	0.000*
AMA †	0 (0)	60 (73.2)	0.000*
Anti-3E †	1 (2.3)	67 (81.7)	0.000*
Anti-SP100 †	1 (2.3)	11 (13.4)	0.056
Anti-PML †	0 (0)	6 (7.3)	0.091
Anti-gp210 ^†^	0 (0)	21 (25.6)	0.000*

*: P < 0.05

†: Forty-four patients in the SS group underwent the autoimmune liver disease test.

ANA, antinuclear antibody; AMA, anti-mitochondrial antibody; ACA, anti-centromere antibody; SS, Sjögren’s syndrome; PBC, primary biliary cholangitis.

### Logistic regression analysis for laboratory risk factors for PBC in patients with SS

**Fig. 1:**
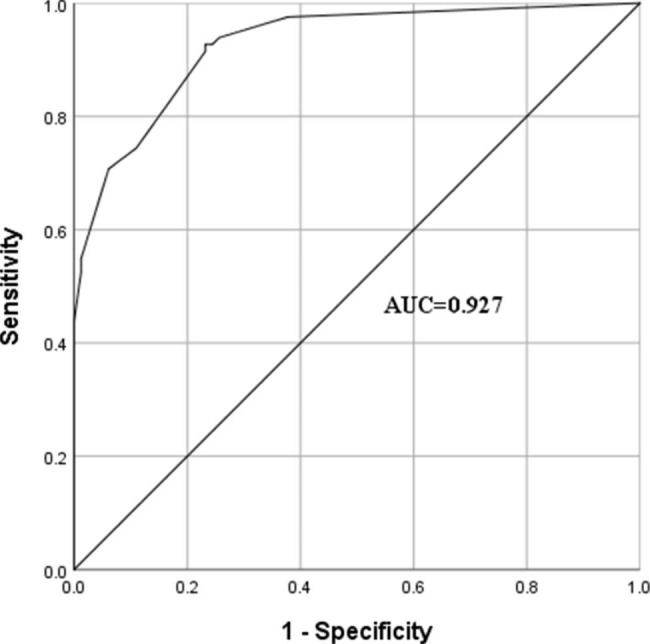
Results of receiver operating characteristic (ROC) analysis of the logistic regression model. AUC: area under the ROC curve

Excluding PBC-specific indices such as liver function, AMA, AMA-M2, anti-3E antibody, and anti-gp210, the statistically significant indices in the routine laboratory blood tests of patients with SS were selected as independent variables, and the presence of coexistent PBC was the dependent variable. Logistic regression analysis showed that elevated IgM levels, high ANA titre (> 10,000), cytoplasmic pattern, and ACA were independent risk factors for PBC complications (Table 4). The AUCs for IgM, ACA, ANA titre, and cytoplasmic pattern were 0.738 (95% confidence interval [CI], 0.660–0.816), 0.628 (95% CI, 0.542–0.714), 0.683 (95% CI, 0.600–0.765) and 0.835 (95% CI, 0.770–0.901), respectively. The AUC result using the four combined indices was 0.927 (95% CI, 0.889–0.965), as shown in Fig. 1.

**Table 4:** Multivariable logistic regression analysis for laboratory risk factors for PBC in patients with SS

Variables	B	S.E	OR	OR (95% CI)	P value
Elevated IgM	1.224	0.598	3.401	1.053–10.989	0.041
ANA title > 1:10000	1.582	0.592	4.863	1.524–15.521	0.008
Cytoplasmic pattern	1.184	0.206	3.269	2.182–4.898	0.000
ACA	2.250	0.737	9.488	2.236–40.255	0.002

OR, odds ratio; CI, confidence interval; Ig, immunoglobulinANA, antinuclear antibody; PBC, primary biliary cholangitis; SS, Sjögren’s syndrome; ACA, anti-centromere antibody.

## Discussion

The liver is an exocrine gland that is frequently involved in SS, and PBC is an autoimmune liver disease that is most frequently associated with SS [13]. Our study found that patients with liver injury caused by SS combined with PBC were more prone to cirrhosis, consistent with previous reports [3, 14]. Apart from conventional factors such as increased levels of liver enzymes and PBC-specific autoantibodies, some other laboratory risk factors of PBC in patients with SS, including elevated IgM levels, ACA positivity, high ANA titre, and cytoplasmic ANA pattern, were shown in this study. These results may aid in the early screening and diagnosis of PBC in patients with SS, especially for patients in the early stage of PBC without elevated ALP levels or those with negative AMA.

We found that the elevated IgM level was an independent risk factor for SS in patients with PBC, suggesting that clinicians should pay attention to IgM at an early stage of the disease. IgM is the primary immunoglobulin involved in the early immune response, and in patients with PBC, it is synthesized at high levels through T and B cell activation, reflected in higher serum concentrations [15–19]. Although there is a disorder in the peripheral B cell population of patients with SS [20], the abnormality of IgM mainly exists in the labial gland tissue [21], while immunological dysregulation in serum is related to IgG-producing clones rather than IgM-producing clones [22]. In addition, SS-associated autoantibodies (anti-SSA and anti-SSB) are reportedly IgG1-dominant [23, 24]. However, the specific AMAs of PBC are predominantly IgG2 and IgG3 [25, 26]. The present study found that in patients with SS + PBC, the levels of IgG3, IgG3/IgG, IgG2, and IgG2/IgG were higher compared to those in patients with SS; however, the levels of IgG1/IgG were lower in patients with SS + PBC than those in patients with SS. We speculated that there was a switch between immunoglobulin subtypes in the combined group, which may be involved in the pathogenesis of comorbidities.

ACA has also been detected in patients with various autoimmune diseases. Previous studies reported that 4.7% of patients with SS and 9–30% of patients with PBC were ACA-positive, and ACA-positive patients had common clinical features [27–30]. This study found that 31.1% of patients with SS + PBC were ACA-positive, which was higher than 6.1% in the SS group. This result was consistent with the literature that reported ACA as a marker of PBC [27, 31], which of diagnostic and prognostic relevance in PBC, particularly in AMA negative cases [30]. However, both groups had a similar prevalence of anti-SSA, anti-SSB, and anti-Ro52 antibodies, which was most likely attributable to SS.

ANA is a standard test used for the differential diagnosis of autoimmune diseases [32]. To the best of our knowledge, the cytoplasmic pattern of ANA indicates the existence of AMA, thus favouring the diagnosis of PBC [32, 33]. It has been reported that 59% of patients with SS display cytoplasmic pattern positivity, and patients with autoimmune disease and cytoplasmic pattern positivity exhibit hepatic involvement [34]. Our study found that 85.4% of patients with SS + PBC had a cytoplasmic pattern, which was similar to the 62.2–85.7% of patients with PBC reported in the literature [33, 35] but higher than the 57.14% (4/13) of patients with SS + PBC in the literature [35]. This may be due to the low case numbers in the literature, and the characteristic pattern of AMA (cytoplasmic granular type AC-21) was not separated from other cytoplasmic patterns in our study, which might have led to an overestimation of the results. Additionally, our study found a higher frequency of the centromeric and nuclear membranous patterns in SS + PBC patients, which is consistent with other findings [36, 37]. In future research, investigating the clinical, diagnostic and prognostic significance of different ANA in PBC, in particular the difference with other antinuclear autoantibodies such as antibodies to speckled protein as recently summarized by Granito et al. [38], will be an important direction.

According to international recommendations, the ANA titre is mainly used to diagnose autoimmune diseases [39]. No difference in ANA titres between SS and SS with PBC was reported in the literature [14]. However, our study found that the ANA titre (≥ 1:10000) of patients with SS + PBC was higher than that of those with SS. Previous studies reported that the ANA titres of 26.9% (83/309) of patients with SS and 40.1% (55/137) of patients with PBC were higher than 1:1000 [33, 34]. Yang et al. Reported that patients with PBC had higher ANA titres [40]. We speculated that the concomitant occurrence of SS and PBC might enhance the immune response, leading to higher antibody positivity.

In routine clinical practice, ALT and AST are the most frequently assessed liver enzymes, whereas ALP and GGT are less frequently available [41]. In addition, because of the high cost and unavailability in many clinics and hospitals, laboratory tests for autoimmune liver disease-specific autoantibodies cannot be widely applied to every patient with SS. These risk factors have great significance in reminding physicians to use further specific investigations for early PBC detection and diagnosis in patients with SS.

Our study has some limitations. This was a retrospective single-centre study. Our study did not record the response to treatment in either group, and the clinical data were not strictly matched, which may have led to deviations in the results.

## Conclusions

Patients with SS complicated with PBC had characteristics different from those with SS alone. In addition to higher levels of liver enzymes, higher frequencies of PBC-associated autoantibodies, elevated IgM levels, positive ACA, and high ANA titre with cytoplasmic pattern also indicated the presence of PBC in patients with SS. For patients with SS with the above risk factors and without elevated liver enzyme levels in the early stage, PBC-related investigations are particularly important for early screening and intervention to reduce the incidence of liver cirrhosis and therefore should be actively performed.

## Data Availability

Data relevant to the study are included in this published article. Additional data supporting the study findings are available from the corresponding author on reasonable request.

## Abbreviations

ACA: Anti-centromere antibody
ALP: Alkaline phosphatase
ALT: Alanine aminotransferase
AMA: Anti-mitochondrial antibody
ANA: Antinuclear-nuclear antibody
Anti-LKM: Anti-liver kidney microsomeantibody
ASMA: Anti-smooth muscle antibody
AST: Aspartate aminotransferase
AUC: Area under the curve
CI: Confidence interval
DBIL: Direct bilirubin
ESR: Erythrocyte sedimentation rate
GGT: γ-glutamyl transferase
IIF: Indirect immunofluorescent
IgG: Immunoglobulin G
IgM: Immunoglobulin M
ILD: Interstitial lung disease
IQR: Interquartile range
LIA: Line immunoassay
PBC: Primary biliary cholangitis
RA: Rheumatoid arthritis
RF: Rheumatoid factor
ROC: Receiver operating characteristic
SLE: Systemic lupus erythematosus
SD: Standard deviation
SS: Sjögren’s syndrome
TBIL: Total bilirubin
WBC: White blood cell
